# The GPCR membrane receptor, DopEcR, mediates the actions of both dopamine and ecdysone to control sex pheromone perception in an insect

**DOI:** 10.3389/fnbeh.2014.00312

**Published:** 2014-09-12

**Authors:** Antoine Abrieux, Line Duportets, Stéphane Debernard, Christophe Gadenne, Sylvia Anton

**Affiliations:** ^1^Neuroéthologie-RCIM, INRA/Université d’Angers, UPRES-EA 2647 USC INRA 1330, SFR QUASAV 4207Beaucouzé, France; ^2^Institut d’Ecologie et des Sciences de l’Environnement IEES Paris, Université Pierre et Marie Curie, UMR 7618Paris, France; ^3^Service d’Enseignement de Biologie Animale, Université Paris-SudOrsay, France

**Keywords:** ecdysone, dopamine, antennal lobe, insect, wind tunnel, GPCR

## Abstract

Olfactory information mediating sexual behavior is crucial for reproduction in many animals, including insects. In male moths, the macroglomerular complex (MGC) of the primary olfactory center, the antennal lobe (AL) is specialized in the treatment of information on the female-emitted sex pheromone. Evidence is accumulating that modulation of behavioral pheromone responses occurs through neuronal plasticity via the action of hormones and/or catecholamines. We recently showed that a G-protein-coupled receptor (GPCR), AipsDopEcR, with its homologue known in Drosophila for its double affinity to the main insect steroid hormone 20-hydroxyecdysone (20E), and dopamine (DA), present in the ALs, is involved in the behavioral response to pheromone in the moth, *Agrotis ipsilon*. Here we tested the role of AipsDopEcR as compared to nuclear 20E receptors in central pheromone processing combining receptor inhibition with intracellular recordings of AL neurons. We show that the sensitivity of AL neurons for the pheromone in males decreases strongly after AipsDopEcR-dsRNA injection but also after inhibition of nuclear 20E receptors. Moreover we tested the involvement of 20E and DA in the receptor-mediated behavioral modulation in wind tunnel experiments, using ligand applications and receptor inhibition treatments. We show that both ligands are necessary and act on AipsDopEcR-mediated behavior. Altogether these results indicate that the GPCR membrane receptor, AipsDopEcR, controls sex pheromone perception through the action of both 20E and DA in the central nervous system, probably in concert with 20E action through nuclear receptors.

## Introduction

In vertebrates, modulation of behavioral output occurs through neuronal plasticity, and involves both neuromodulators such as biogenic amines and endocrine factors (Hull et al., 2004; Hull, 2011). Also in arthropods, both hormones and neuromodulators are involved in behavioral plasticity by mediating structural and physiological changes (Walikonis et al., 1991; Linn et al., 1992; Sigg et al., 1997; Lehman et al., 2000; Jarriault et al., 2009). 20-hydroxyecdysone (20E), the major insect steroid hormone, is mainly known to modulate developmental processes, adult physiology and sexual behavior by interacting with a nuclear complex composed of the ecdysone receptor (EcR) and its partner ultraspiracle (USP) and thus eliciting genomic actions (Bigot et al., 2012; Fahrbach et al., 2012; Duportets et al., 2013). On the other hand biogenic amines such as dopamine (DA), octopamine, serotonin or tyramine are well described to orchestrate a broad range of physiological functions when binding with a wide panel of membrane-coupled receptors (Roeder, 2005; Lange, 2009; Duportets et al., 2010; Van Swinderen and Andretic, 2011; McQuillan et al., 2012). However, crosstalk between these different modulators has only been studied in a few invertebrate species so far, particularly with regard to their effects on behavior (Kravitz, 2000; Schulz et al., 2002; Bloch and Meshi, 2007; Gruntenko et al., 2007; Jarriault et al., 2009; Rauschenbach et al., 2012; Geddes et al., 2013). Uncommonly 20E can also have non-genomic effects through interaction with DopEcR, a double-affinity membrane receptor belonging to the G-protein-coupled receptor (GPCR) superfamily, which also binds DA, and identified originally in *Drosophila melanogaster* (Srivastava et al., 2005; Evans et al., 2009). This double-affinity receptor differs from other well-known specific DA receptors (Dop-R), which have been found in many insect species (Blenau and Baumann, 2001).

In moths, males use female-emitted pheromones to find their mating partners. In the male noctuid moth, *Agrotis ipsilon*, several aspects of neuronal plasticity have been revealed, which are at the origin of the modulation of behavioral pheromone responses (Anton et al., 2007). Newly emerged males are sexually immature and do not respond behaviorally to the female-produced sex pheromone. 3–5 days after emergence, males become sexually mature and are highly attracted by the sex pheromone (Gadenne et al., 1993). This increase in pheromone response with age is paralleled with an increase in the sensitivity of neurons in the primary olfactory center, the antennal lobe (AL; Anton and Gadenne, 1999). Hormones such as juvenile hormone (JH) and 20E, as well as catecholamines such as octopamine have been shown to be involved either alone or in interaction in this behavioral and central nervous olfactory plasticity (Anton and Gadenne, 1999; Jarriault et al., 2009; Duportets et al., 2013).

Recently we identified DopEcR in *A. ipsilon*, AipsDopEcR, and demonstrated that it is involved in the behavioral response to sex pheromone (Abrieux et al., 2013). We found this receptor predominantly expressed in the ALs and mushroom bodies (MBs), both structures involved in the central processing of the sex pheromone. Moreover, using RNA interference (RNAi) we reported that AipsDopEcR silencing drastically inhibited the behavioral response to the sex pheromone (Abrieux et al., 2013). However, it is unknown so far if the effect of DopEcR inhibition on pheromone-guided behavior is mediated by a modulation of central nervous responses to the sex pheromone. To further unveil the putative role of this GPCR in the modulation of pheromone signal integration, we used intracellular recordings of AL neurons in AipsDopEcR-silenced moths. We compared response thresholds for the sex pheromone between AipsDopEcR-dsRNA-injected and control males.

Moreover the respective roles of 20E and DA in the control of behavioral responses to pheromone through their action on AipsDopEcR remain unknown. Concerning ecdysteroids, recent data show that 20E injection in immature males can increase both EcR and USP expression, concomitantly with the behavioral sensitivity of males for the sex pheromone (Duportets et al., 2013). On the other hand, injection of cucurbitacin B (CurB), an antagonist of 20E able to interact with the EcR/USP complex, inhibited the behavioral pheromone response in *A. ipsilon* (Duportets et al., 2013). Although another catecholamine, octopamine, was found to be necessary to elicit sexual attraction behavior in *A. ipsilon* (Jarriault et al., 2009), nothing is known concerning the role of DA. We investigated the contribution of each ligand within the DopEcR pathway with behavioral tests in a wind tunnel. For this we tested the role of each ligand alone on pheromone responses first in control males, and then in males for which the DopEcR and/or the EcR/USP pathway were blocked by RNAi or CurB respectively to dissociate actions mediated by the two pathways and the two ligands. We also determined pheromone response thresholds of AL neurons in males injected with CurB to compare the roles of EcR/USP with DopEcR in central pheromone processing.

Our results show that inhibition of both AipsDopEcR and the EcR/USP complex reduce the sensitivity of AL neurons to sex pheromone. The control of sex pheromone perception by AipsDopEcR seems to be mediated by the combined action of both ligands, 20E and DA.

## Materials and methods

### Insects

Experiments were performed with adults of *A. ipsilon* originating from a laboratory colony in Bordeaux. The colony was based on field catches in southern France and wild insects are introduced each spring. The animals were reared on an artificial diet (Poitout and Buès, 1974) in individual cups until pupation. Pupae were sexed and males and females were kept separately in an inversed light/dark cycle (16 h light: 8 h dark photoperiod, with scotophase starting at 10 am) at 22°C. Newly emerged adults were removed from the hatching containers every day, and were given access to a 20% sucrose solution *ad libitum*. The day of emergence was considered as day 0.

### dsRNA synthesis

AipsDopEcR-dsRNA (586 bp) and LacZ-dsRNA (372 bp) preparation was performed as previously described (Abrieux et al., 2013). Briefly a PCR was performed on 1 μL of plasmid (50 ng/mL) with specific primers of each target gene DopEcR T7 dir/DopEcR T7 rev and LacZ T7 dir/LacZ T7 rev. PCR products were purified with NucleospinH extract II kit (Macherey Nagel) and quantified by nanodrop. Then a transcription reaction was performed using T7 RNA polymerase enzyme and obtained dsRNAs were precipitated with LiCl. Samples were denaturated followed by a rehybridization step at room temperature. Finally, dsRNA integrity was checked by loading on agarose gel. Before injection, dsRNA was diluted at 0.5 μg/μL in saline solution. One-day-old adult males were injected with 1 μg dsRNA into the abdomen in order to perform intracellular recordings and behavioral tests at day-5. For both series of experiments, control groups consisted of bacterial LacZ-dsRNA-, Ringer-, and non-injected males.

### Chemicals

Pheromone stimulation was performed with an artificial pheromone blend containing (Z)-7-dodecen-1-yl acetate (Z7–12:OAc), (Z)-9-tetradecen-1-yl acetate (Z9–14:OAc), and (Z)-11-hexadecen- 1-yl acetate (Z11–16:OAc) (Sigma Aldrich, Saint-Quentin Fallavier, France) at a ratio of 4:1:4 (Picimbon et al., 1997; Gemeno and Haynes, 1998), which has been used successfully in field trapping experiments (Causse et al., 1988). 1 ng of the pheromone blend was used for all behavioral tests as this dose was shown to give suboptimal responses (around 50% responses) with sexually mature virgin males (Barrozo et al., 2010), in order to allow for an increase in response rates with certain treatments. For electrophysiological experiments, the pheromone blend was diluted in decadic steps in hexane resulting in doses from 1 pg to 100 ng applied on a filter paper introduced in a Pasteur Pipette.

20-Hydroxyecdysone was a gift from Pr. René Lafont (Pierre et Marie Curie University, Paris, France) and the antagonist of the 20E/EcR/USP complex, CurB, was purchased from Sigma Aldrich. Stock solutions of 20E and CurB were prepared in ethanol at a concentration of 10^−2^ M, then stored at −20°C. For experiments, the stock solutions were diluted to 10^−5^ M in a NaCl (145 mM) solution. DA was purchased from Sigma Aldrich, and was used at a dilution of 15 μg/μl. A new solution was prepared each day of experiments.

### Injection treatments

All injections were performed in the abdomen 30–90 min before the onset of the scotophase. For AipsDopEcR-dsRNA or control solutions (LacZ-dsRNA or Ringer), 1-day-old males were injected with 2 μl of a solution at 0.5 μg/μl as described previously (Abrieux et al., 2013). For DA, 5-day-old males received an injection of 2 μl of a solution of 15 μg/μl. DA injected in the moth hemocoel has previously been shown to result in increased brain levels of DA (Linn et al., 1994). For 20E and CurB, 5-day-old males received an injection of 2 μl of a 10^−5^ M solution as described previously (Duportets et al., 2013). Control experiments were performed by injection of 2 μl Ringer solution or ethanol solutions diluted as for DA and CurB solutions.

### Electrophysiological experiments

The responses of AL neurons from AipsDopEcR-dsRNA-, CurB- or control-injected males were evaluated at day-5, using intracellular recordings. Control and treated males were used for electrophysiological experiments between 4 h and 7 h after the beginning of the scotophase. Moths were immobilized in a cut disposable pipette tip, the head capsule was opened, and tissue overlaying the brain removed, as described previously (Gadenne and Anton, 2000). Standard intracellular recording techniques were used (Christensen and Hildebrand, 1987). A KCl-filled glass microelectrode was placed close to the cumulus, the biggest part of the macroglomerular complex (MGC) within the AL of the moths as previously described (Jarriault et al., 2009). A 200 ms pheromone stimulus was introduced in a constant airstream (5 mls^−1^) with a stimulation device (CS55 Syntech, Kirchzarten, Germany) when intracellular contact had been established. Each neuron was stimulated using a range of 9 pheromone doses from 0.01 pg to 1 μg starting with low doses and with inter-stimulus intervals of at least 10 s. A Pasteur pipette containing a filter paper with the solvent (hexane) was used as a control. Data were registered, and analyzed off-line using Autospike 32 software (Syntech, Kirchzarten, Germany). For the analysis of neuron thresholds, spikes were counted manually, and net-spikes were calculated from the number of spikes during a period after the stimulus minus the number of spikes counted during the same preceding period (representing spontaneous activity). The time interval was chosen to include the excitatory response in the majority of the responses to the stimuli for each neuron. A neuron was classified as responding to a stimulus when the odor response exceeded the hexane response by at least 20% and the lowest dose eliciting a response was defined as the threshold dose. Data are presented as cumulative threshold curves as a function of stimulus dose threshold distributions. The responses of AL neurons to sex pheromone were evaluated by comparing the proportion of neurons responding at different thresholds from the males of different groups. To check for statistical differences among treatments, a *R* X *C* test of independence was performed by using a *G*-test and applying the Williams’s correction (Sokal and Rohlf, 1995). In addition, *post*-*hoc* comparisons were carried out and the experimental-wise error rate was corrected by means of the Dunn-Sidák method (Sokal and Rohlf, 1995).

### Design of behavioral experiments

**Figure 1:**
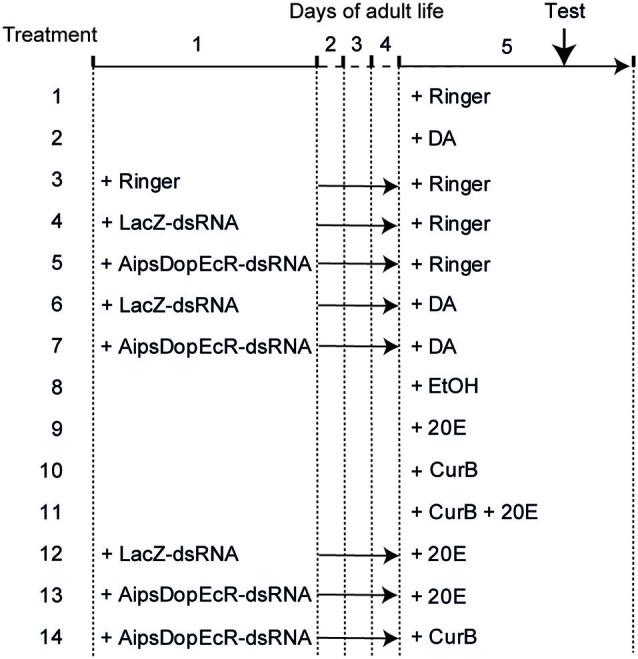
**Design of experiments**. Treatments were performed on 1-day-old and 5-day-old *A. ipsilon* males. Test: behavioral test in wind tunnel. DA: Dopamine; 20E: 20-hydroxyecdysone; CurB: cucurbitacine.

To analyze the possible action of 20E and DA on AipsDopEcR, different treatments were performed on *A. ipsilon* males (Figure 1), and their responses to the sex pheromone were tested in the wind tunnel. For this we injected DA or 20E, the potential inhibitors of their receptors (AipsDopEcR-dsRNA for DA and 20E, and/or CurB for 20E), and a combination of both.

First, a control experiment was performed by testing the behavioral responses of 5-day-old Ringer-injected males (treatment 1). Then the possible involvement of DA was evaluated by testing the responses to sex pheromone of 5-day-old DA-injected males (treatment 2). Next a second control experiment consisted in testing the responses of 5-day-old males injected with Ringer, previously injected at day-1 also with Ringer (treatment 3). A third experiment consisted in testing the responses of 5-day-old males injected with Ringer previously injected at day-1 with LacZ-dsRNA as control or AipsDopEcR-dsRNA (treatments 4 and 5). The next experiment consisted in testing the responses of 5-day-old males injected with DA previously injected at day-1 with LacZ-dsRNA as control or AipsDopEcR-dsRNA, to test whether an excess of DA could restore the expected response inhibition induced by AipsDopEcR silencing (treatments 6 and 7) (Figure 1).

Similarly, although we already showed its absence of effect (Duportets et al., 2013), the possible involvement of 20E was again checked by testing the responses to sex pheromone of 5-day-old diluted ethanol-injected males as control or 20E-injected males (treatments 8 and 9). Another experiment consisted in analyzing the responses of 5-day-old CurB and CurB + 20E-injected males, to test whether 20E could restore the expected response inhibition induced by CurB (Duportets et al., 2013) (treatments 10 and 11). Next we analyzed the responses of 5-day-old males injected with 20E, previously injected at day-1 with LacZ-dsRNA as control or AipsDopEcR-dsRNA to test whether 20E could restore the inhibition induced by AipsDopEcR silencing (treatments 12 and 13) (Figure 1).

Last, the effect of both AipsDopEcR silencing and USP/EcR inhibition was analyzed by testing the responses of 5-day-old males injected with CurB, previously injected at day-1 with AipsDopEcR-dsRNA (treatment 14) (Figure 1).

### Wind tunnel experiments

Behavioral tests were performed using a 2 m-long flight tunnel during the middle of the scotophase (4–7 h after lights off) under red light illumination as previously described (Barrozo et al., 2010). Environmental conditions during the bioassay were held constant: 22°C, 50 ± 10% relative humidity, wind speed of 0.3 ms^−1^. A cage containing a single experimental male was introduced in the wind tunnel. After 30 s, during which the male adjusted to the airflow, a filter paper containing the stimulus was placed 160 cm upwind from the cage. The behavior of the moths was observed for 3 min, and partial flight, complete flight and landing on the pheromone source were considered as an oriented response. We also noted the latency of each oriented response. All experiments were performed double-blind to avoid partial observations. Each day of experiments, different groups of males were tested including at least one group of males that were expected to show a high response level to avoid experimental bias. Statistical differences (*P* < 0.05) were evaluated using a *R* X *C* test of independence using a *G*-test and applying the Williams’ correction (Sokal and Rohlf, 1995). In addition, individual *post hoc* comparisons were carried out and the experimental-wise error rate was adjusted by using the Dunn–Sidák method (Sokal and Rohlf, 1995). Differences in pheromone response delays between groups were evaluated using the non parametric Kruskal-Wallis test followed by Mann-Whitney tests for pairwise comparisons (*P* < 0.05) with GraphPad Prism version 6 (GraphPad Sofware).

## Results

### Effect of AipsDopEcR silencing or CurB injection on AL neuron responses

**Figure 2:**
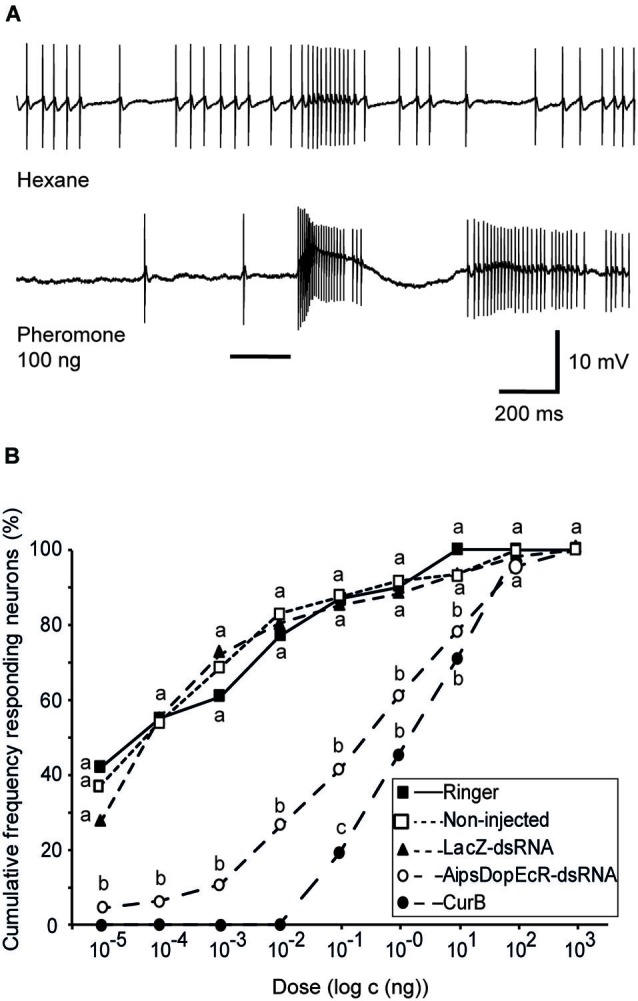
**Effects of AipsDopEcR-dsRNA and CurB injection on response thresholds of AL neurons in 5-day old *A. ipsilon* males. (A)** Example of intracellular recording traces of an AL neuron from an AipsDopEcR-injected male stimulated with hexane and the sex pheromone. Note the excitatory response followed by inhibition. Bar beneath recording indicates stimulus duration (200 ms). **(B)** Cumulative percentage of tested AL neurons responding to the pheromone blend at different thresholds. *N* = 31, 49, 63, 46, and 42 neurons for Ringer-, non-injected, LacZ-dsRNA-, CurB-, and AipsDopEcR-dsRNA-injected males respectively. Values with the same letters are not significantly different between treatments (*G*-test, *P* < 0.05). CurB: cucurbitacine.

Intracellularly recorded AL neurons showed excitatory responses to the pheromone, followed in most cases by an inhibitory period, which is characteristic for MGC projection neuron responses (Jarriault et al., 2009; Figure 2A).

Intracellular recordings were performed on AL neurons of two control groups (48 AL neurons of non-injected, and 61 AL neurons of dsRNA-LacZ-injected males) and their response thresholds for the sex pheromone was compared to that of 46 AL neurons from individuals injected with AipsDopEcR-dsRNA. Response threshold distributions of AL neurons to the sex pheromone were shifted significantly between AipsDopEcR-dsRNA-injected and control males (Figure 2B). AL neurons in dsRNA-DopEcR-injected males have a cumulative threshold curve, which is shifted to higher doses as compared to dsRNA-LacZ-injected or non-injected 5 day-old males. No statistical difference in the threshold curves was observed between AL neurons of non-injected and dsRNA-LacZ-injected males (*G* = 10.19; *df* = 12; *p* = 0.6). In contrast, the threshold curve obtained from neurons recorded from AipsDopEcR-dsRNA-injected males was significantly different from threshold curves of the respective control groups, non-injected and dsRNA-LacZ-injected (*G* = 39.79; 43.75; *df* = 7; *p* < 0.0001) (Figure 2B).

Intracellular recordings were also performed on AL neurons of CurB-injected males (42 neurons). As for neurons of AipsDopEcR-dsRNA-injected males, response threshold distributions of AL neurons from CurB-injected males were shifted to higher doses as compared to Ringer-injected males (31 neurons) (Figure 2B). The threshold curve of CurB-injected males was significantly different from those of the Ringer control group (*G* = 63.47; *df* = 5; *p* < 0.0001) and different from that of AipsDopEcR-dsRNA-injected males only for the pheromone dose of 100 pg (*G* = 5.15; *df* = 1; *p* = 0.023) (Figure 2B).

### The role of DA and 20E in AipsDopEcR-mediated behavior

**Figure 3:**
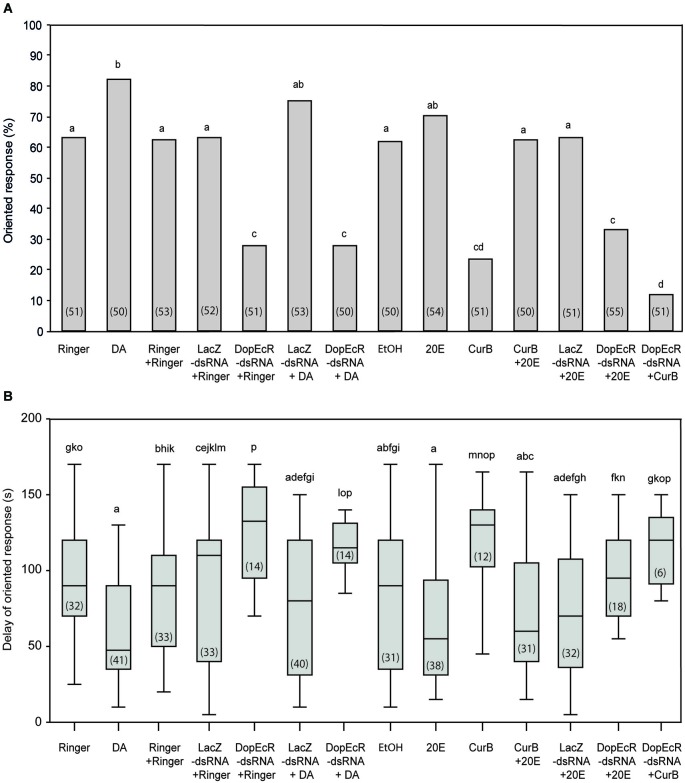
**Effects of individual or combined DA, 20E, AipsDopEcR-dsRNA, and CurB treatments on upwind flight behavior of *A. ipsilon* males to the sex pheromone in a wind tunnel**. **(A)** Oriented response. **(B)** Delay of response for males that performed an oriented response (means ± SD). DA: Dopamine; 20E: 20-hydroxyecdysone; CurB: cucurbitacine. Bars with same letters are not significantly different (**A**: *G*-test; **B**: Kruskal-Wallis test followed by a Mann-Whitney test for pairwise comparisons; *P* < 0.05). Numbers in brackets indicate numbers of tested males **(A)** and males that performed an oriented response **(B)**.

The percentage of oriented responses of DA-injected males to the sex pheromone (treatment 2; 82%) was significantly higher than that of Ringer-injected males (treatment 1; 63%) (*G* = 4.65; *p* = 0.030) (Figure 3A) and the response delays were among the shortest observed (Figure 3B).

In order to evaluate DA effects mediated by AipsDopEcR, we tested the responses to the sex pheromone of AipsDopEcR-dsRNA + DA-injected males, which were compared with that of AipsDopEcR-dsRNA + Ringer-injected males, and with the responses of control-injected males (Ringer + Ringer-, LacZ-dsRNA + Ringer- and LacZ-ds RNA + DA-injected males: treatments 3, 4, and 6) (Figure 3A). The percentage of responses of AipsDopEcR-dsRNA + DA-injected males (treatment 7; 28%) was not different from that of AipsDopEcR-dsRNA + Ringer-injected males (treatment 5; 28%) (*G* = 0; *p* = 1) but significantly different from responses of the three control-injected groups (*G* = 12.27, *p* = 0.0004, *G* = 13.01; *p* = 0.0003, *G* = 23.85; *p* = 0.0001) (Figure 3A). Responses of LacZ-dsRNA + DA-injected males were not significantly different from that of DA-injected males (*G* = 0.64; *p* = 0.42) but also not different from LacZ + Ringer-injected males (*G* = 1.76; *p* = 0.18). Confirming results from an earlier study (Abrieux et al., 2013), the response of AipsDopEcR-dsRNA + Ringer-injected males (28%) was statistically different from that of Ringer + Ringer-injected males (treatment 3; 62%) (*G* = 12.27; *p* = 0.0004) (Figure 3A) and from LacZ-dsRNA + Ringer-injected males (*G* = 13.01; *p* = 0.0003). Response delays were among the longest observed for AipsDopEcR-dsRNA + Ringer- and + DA-injected males (Figure 3B). Altogether, this shows that a surplus of DA cannot compensate the strong behavioral decrease induced by AipsDopEcR silencing.

To evaluate the role of 20E as modulator of pheromone-guided behavior, we first tested the behavioral response of 20E-injected males (treatment 9; 70%), which was not significantly different from that of Ringer-injected males (treatment 1; 63%) (*G* = 0.47; *p* = 0.49) or diluted ethanol-injected males (treatment 8) (*G* = 0.80; *p* = 0.37) (Figure 3A). The response delay of 20E-injected males was, however, significantly shorter than in Ringer-injected males (*U* = 916; *p* = 0.001) (Figure 3B). Next we tested the possible role of 20E in the AipsDopEcR- and/or the USP/EcR-mediated modulation. The proportion of oriented responses of CurB-injected males (treatment 10; 24%) was statistically different from that of Ringer-injected males (63%) (*G* = 17.09; *p* = 0.00003). However, the injection of 20E into CurB-injected males completely restored their behavior in terms of response percentage (treatment 11; 62%) (Figure 3A) and response delay (Figure 3B). On the other hand, the percentage of responses of AipsDopEcR-dsRNA + 20E-injected males (treatment 13; 31%) to the sex pheromone were not different from those of AipsDopEcR-dsRNA + Ringer-injected males (*G* = 0.15; *p* = 0.70), but significantly lower than responses in LacZ-dsRNA + 20E-injected males (treatment 12) (*G* = 9.57; *p* = 0.0019) (Figure 3A). However, the response delay of males injected with AipsDopEcR-dsRNA + 20E was significantly reduced as compared with that of singly DopEcR-dsRNA-injected males (*U* = 194.5; *p* = 0.01) (Figure 3B). Altogether, this shows that 20E can compensate for the strong decrease of behavioral responses induced by CurB, an antagonist of USP/EcR receptors, but only reduces the response delay, without restoring the percentage of oriented responses induced by AipsDopEcR silencing.

Lastly, we analyzed the effects of combined AipsDopEcR silencing and USP/EcR inhibition by CurB on sex pheromone responses (Figure 3A). The oriented responses of AipsDopEcR-dsRNA + CurB-injected males (treatment 14; 12%) were even lower and statistically different from those of AipsDopEcR-dsRNA + Ringer-injected males (28%) (*G* = 4.03; *p* = 0.044), but response latencies did not change significantly (*U* = 54.5; *p* = 0.319) (Figure 3B).

## Discussion

### AipsDopEcR and the EcR/USP complex are involved in the modulation of AL neuron sensitivity to sex pheromone

In the present study we show that the inhibition of the behavioral response to sex pheromone previously observed in AipsDopEcR-silenced *A. ipsilon* males using an RNAi approach (Abrieux et al., 2013) might originate from a decrease in pheromone sensitivity at the AL level. Intracellular recordings show that a 1000-fold higher pheromone dose is necessary to elicit a response in neurons of AipsDopEcR-dsRNA*-*injected males as compared to the dose eliciting responses in neurons from control or LacZ-dsRNA-injected males. The low sensitivity of pheromone-responding neurons in AipsDopEcR-dsRNA*-*injected males is consistent with the detection of AipsDopEcR protein in cell bodies of AL neurons and reinforces our hypothesis attributing a role to this receptor in pheromone signal processing (Abrieux et al., 2013). Although we cannot entirely exclude modulation at the peripheral level, the presence of only minute traces of AipsDopEcR in antennal tissue (Abrieux et al., 2013) makes it unlikely that the effect observed within the AL has its origin in modulation of pheromone sensitive receptor neurons.

However, not only inhibition of AipsDopEcR, but also application of the antagonist of the EcR/USP complex, CurB resulted in a similar strong increase in response thresholds. This low level of AL sensitivity in neurons from AipsDopEcR-dsRNA-and CurB-treated males resembles that of immature non-pheromone responding 1-day-old males (Anton and Gadenne, 1999), in which the expression of AipsDopEcR and EcR/USP is naturally low (Abrieux et al., 2013; Duportets et al., 2013). Similarly, we previously found a decrease of AL neuron sensitivity after treatment with mianserin, an antagonist of another GPCR for the catecholamine octopamine, which was shown to be also involved in olfactory plasticity (Jarriault et al., 2009).

### Effects of DA on behavioral responses to pheromone are mediated primarily by AipsDopEcR

According to our results, DA seems to modulate pheromone responses in *A. ipsilon* males, as injection of exogenous DA into sexually mature males led to a significant increase of the oriented upwind flight compared to Ringer-injected individuals. Similarly, another catecholamine, octopamine, was also shown to enhance pheromone responses (Jarriault et al., 2009). An implication of DA in sexual behavior has also been found in other insects. In *D. melanogaster*, for example, DA enhances male-male courtship (Liu et al., 2008), and DA neurons modulate pheromone responses (Keleman et al., 2012). In vertebrates, DA is well known to enhance sexual behavior, contrary to serotonin (Hull et al., 2004).

As injection of exogenous DA into AipsDopEcR-dsRNA-injected males did not restore upwind flight towards the sex pheromone, we conclude that DA affects sexual behavior through AipsDopEcR. Similarly, in *D. melanogaster*, DopEcR, which is expressed in sugar-sensitive gustatory receptor neurons, mediates the effect of L-Dopa feeding to enhance the proboscis extension reflex, by increasing the behavioral sensitivity to sucrose (Inagaki et al., 2012). On the contrary, Drosophila DA neurons were found to control courtship learning through the action of another type of DA receptors, Dop-R1 (Keleman et al., 2012).

### AipsDopEcR and USP/EcR mediate the effects of 20E on behavioral responses to pheromone

In the present study we confirm that the addition of 20E has no effect on the percentage of responding mature males to pheromone, although it has an effect on young immature males as previously described (Duportets et al., 2013; Vitecek et al., 2013). Nevertheless, we found here a reduction in response delay after 20E injection in mature males, indicating that there is still some effect. We also show that 20E is still necessary in mature males to elicit upwind flight behavior, as the injection of CurB, an antagonist of USP/EcR receptors, similarly to the injection of AipsDopEcR-dsRNA, inhibited the response as previously described (Duportets et al., 2010; Abrieux et al., 2013). Thus also the USP/EcR pathway seems to be necessary for the action of circulating 20E on behavioral pheromone responses. Genomic effects on sex pheromone responses based on the interaction between 20E and EcR/USP have been reported previously (Bigot et al., 2012; Fahrbach et al., 2012; Duportets et al., 2013). The fact that 20E-injection restored the decrease of behavior induced by CurB injection indicates that an excess of 20E was probably able to counteract the inhibition of USP/EcR receptors, whereas it only partially restored response delays in AipsDopEcR-dsRNA-injected males and did not influence the percentage of responding males. Also the increased response inhibition of combined treatments with CurB and AipsDopEcR-dsRNA indicates that 20E might act on the behavioral response to sex pheromone via both types of receptors, combining genomic and non-genomic effects. The results from our electrophysiological experiments discussed above support this hypothesis, as inhibition of both receptor types also leads to a strong shift in response thresholds of pheromone-responding AL neurons. Future experiments will have to clarify, however, if these physiological changes in AL neurons can also be counteracted by 20E injection.

## Conclusions

From our results we can conclude that AipsDopEcR mediates action of both DA and 20E to modulate behavioral responses to sex pheromone. The question remains now to understand how this receptor might modulate the sensitivity of AL neurons, via the action of its ligands. Non-genomic effects of 20E have previously been shown to be very fast: ecdysteroids have been shown to rapidly modulate electrical activity in insect neurosecretory cells in the moth *Manduca sexta* (Ruegg et al., 1982), to rapidly depress synaptic efficiency in neuromuscular junctions in crayfish (Cooper and Ruffner, 1998) and in fruitfly larvae (Ruffner et al., 1999; Li et al., 2001). The possible modulatory role of ecdysteroids via DopEcR during processing of sensory information resembles also the well-known non-genomic effects of estrogen on neuronal signaling and memory in vertebrates (Srivastava et al., 2008). There are indications in *D. melanogaster*, that “agonist-specific coupling” exists for different GPCRs, such as for example the D1-like DA receptor DopR99B, where different ligands activate different second messenger pathways, at least for synthetic agonists (Reale et al., 1997). Indeed, pharmacological studies performed in clonal cell lines expressing DopEcR revealed that *Drosophila* DopEcR demonstrates “agonist-specific coupling”: only DA was able to increase cAMP responses whereas 20E activated the MAP-kinase pathway (Srivastava et al., 2005; Evans et al., 2009). However, it was recently shown that *Drosophila* DopEcR could also mediate the action of 20E through cAMP signaling in the adult brain linked with courtship learning (Ishimoto et al., 2013). It is likely that the binding of different agonists to the receptor produces different responses due to the induction of different receptor conformations by the different agonists (Evans et al., 1995; Kenakin, 1995). In *A. ipsilon*, where genetic tools are unavailable, pharmacological experiments on DopEcR expressed in a heterologous system would be needed to reveal the mechanisms of action through which 20E and DA modulate neural and behavioral responses.

## Authors and contributors

Antoine Abrieux, Line Duportets, Stéphane Debernard, Christophe Gadenne and Sylvia Anton designed research. Antoine Abrieux, Christophe Gadenne, and Sylvia Anton performed experiments. Antoine Abrieux, Christophe Gadenne and Sylvia Anton analyzed data. Antoine Abrieux, Line Duportets, Stéphane Debernard, Christophe Gadenne, and Sylvia Anton wrote the manuscript.

## Conflict of interest statement

The authors declare that the research was conducted in the absence of any commercial or financial relationships that could be construed as a potential conflict of interest.
